# Supplementary figures and images for: Biobank-scale characterization of Alzheimer’s disease and related dementias identifies potential disease-causing variants, risk factors, and genetic modifiers across diverse ancestries
Source: medRxiv. 2024 Nov 17:2024.11.03.24313587. Preprint. [Version 4] doi: 10.1101/2024.11.03.24313587 (PMC11601747; doi:10.1101/2024.11.03.24313587)

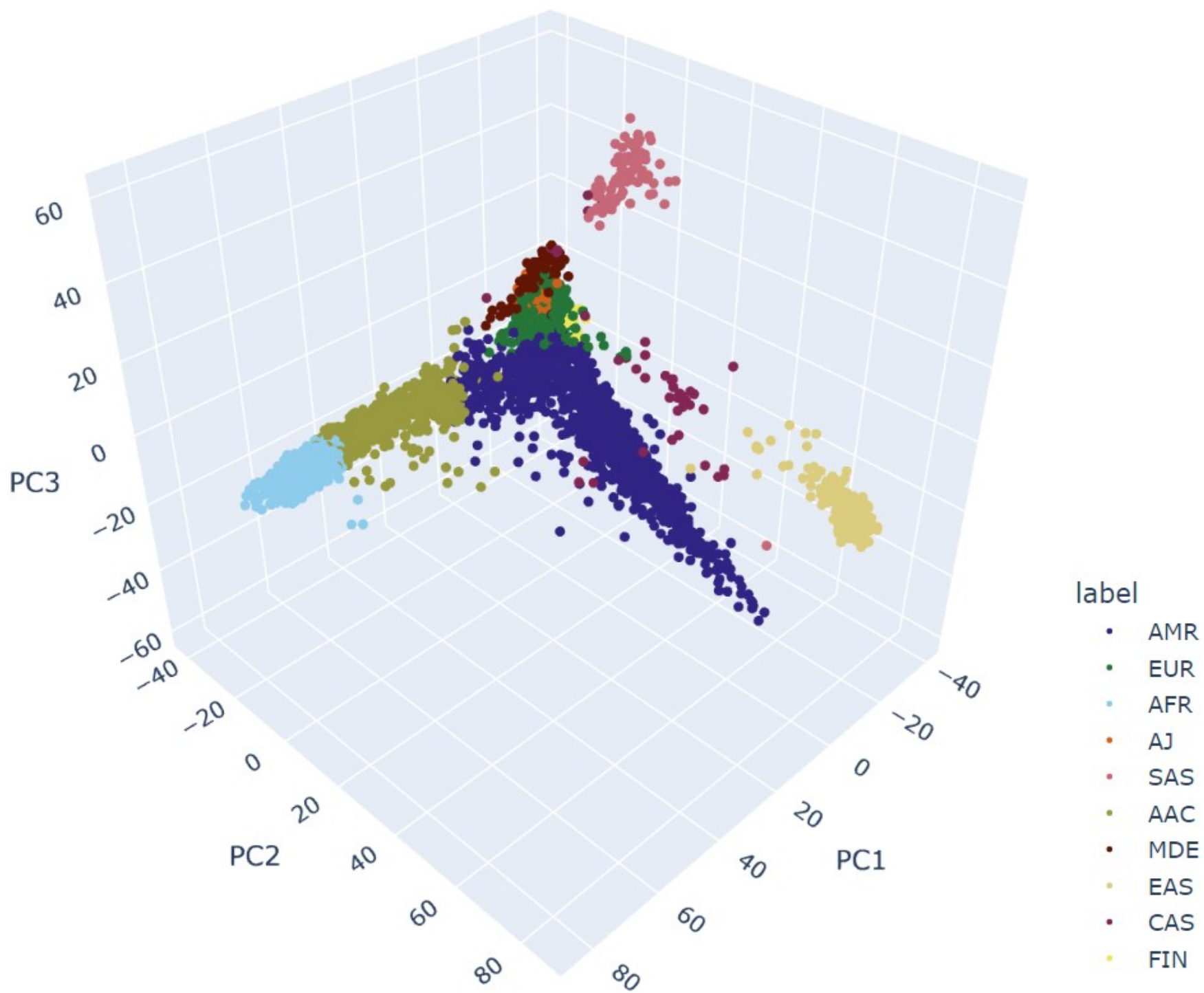

Supplement: Supplement 1 — Supplementary Figure 1- PCA plots in (A) All of Us, (B) UKB, (C) ADSP, (D) AMP PD, and (E) 100 KGP. [file media-1.pdf]

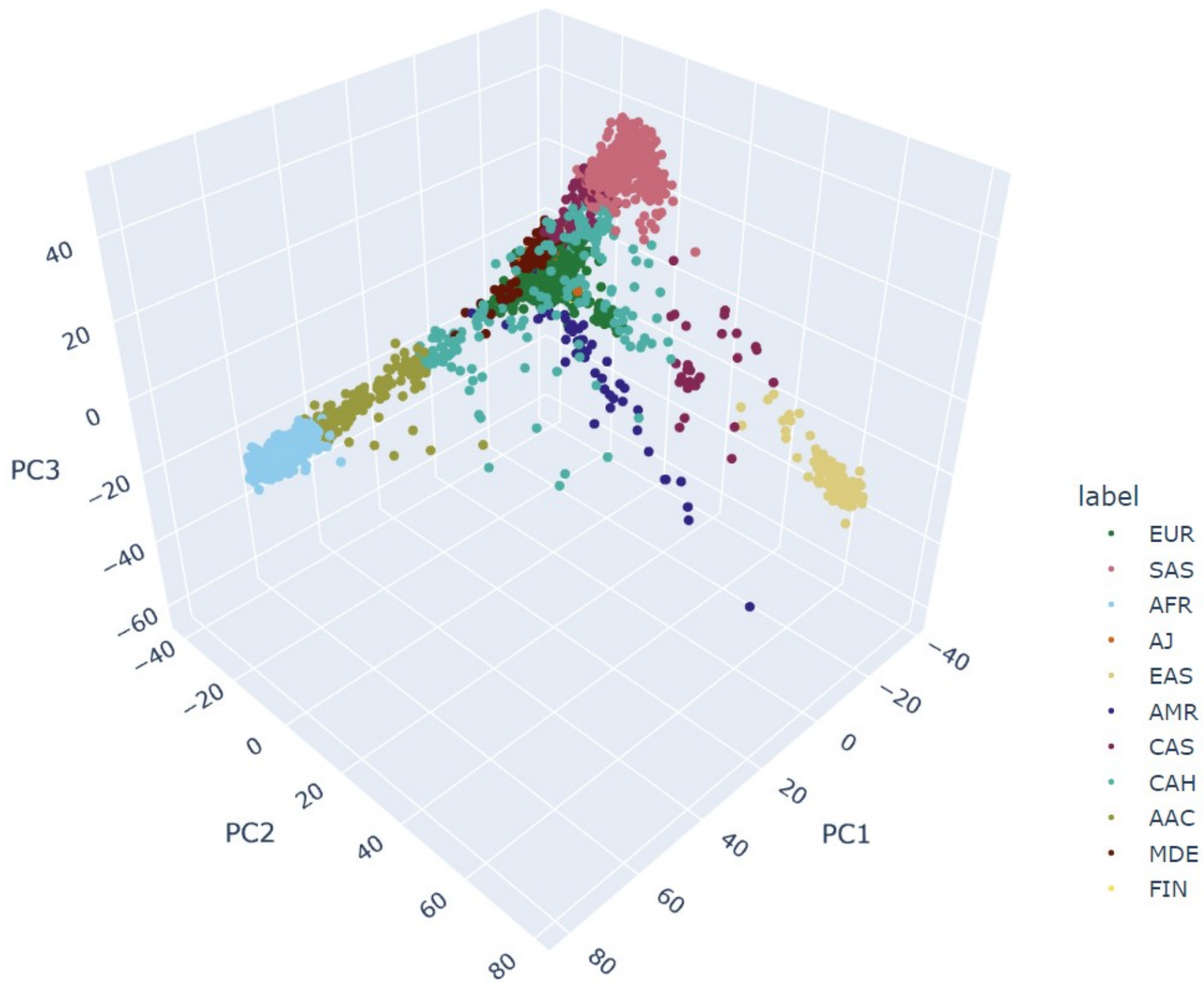

Supplement: Supplement 2 — Supplementary Figure 2- Heatmaps showing the frequencies of all identified variants in the discovery and replication phases across all ancestries in each biobank. [file media-2.pdf]

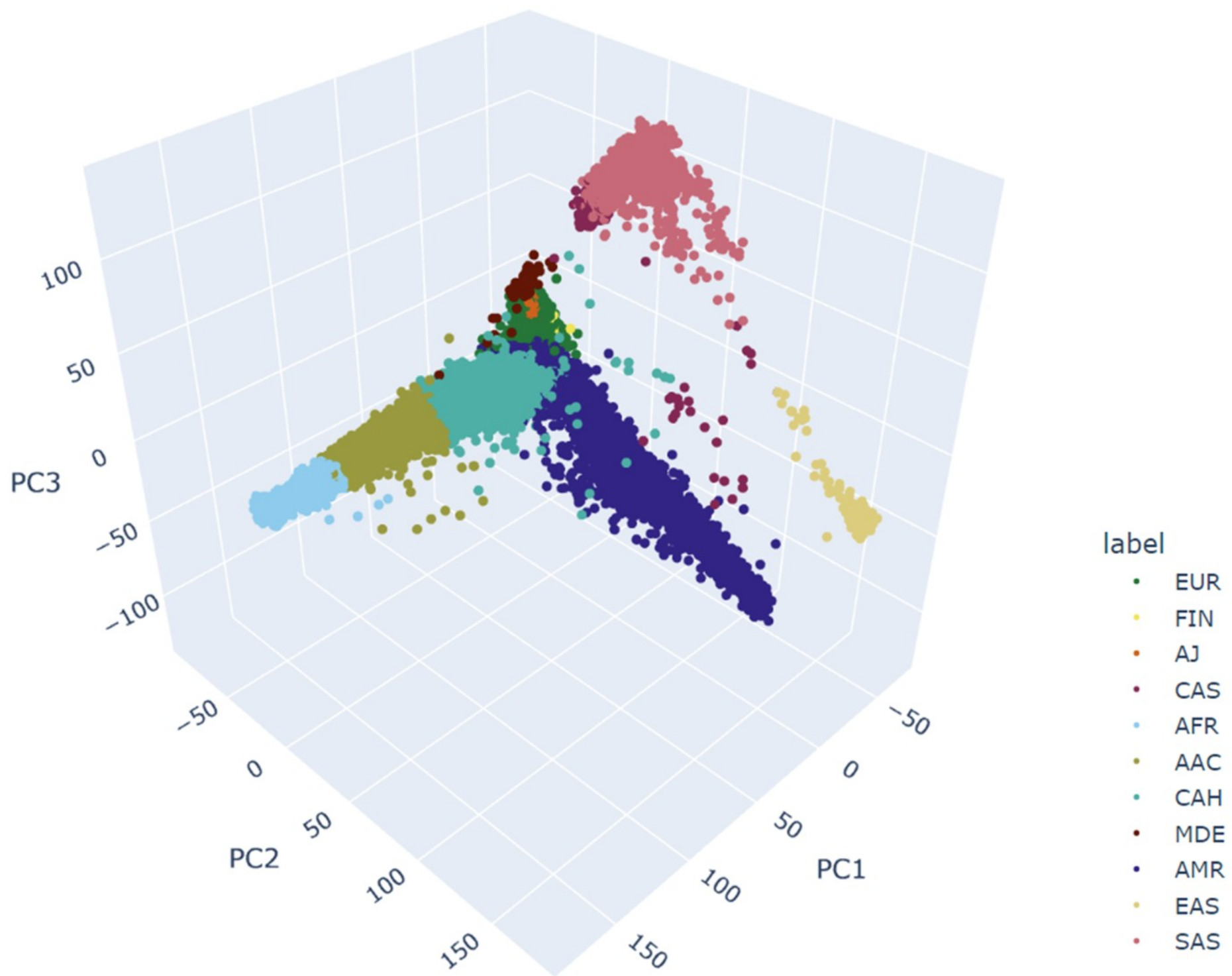

Supplement: Supplement 3 — Supplementary Figure 3- Proportion of APOE genotypes in (A) Alzheimer’s disease, (B) related dementias, and (C) controls across 11 genetic ancestries. Unknown genotypes and those absent across all ancestries were excluded from the analysis. Genotypes and ancestries not available in the 100KGP were also excluded. [file media-3.pdf]

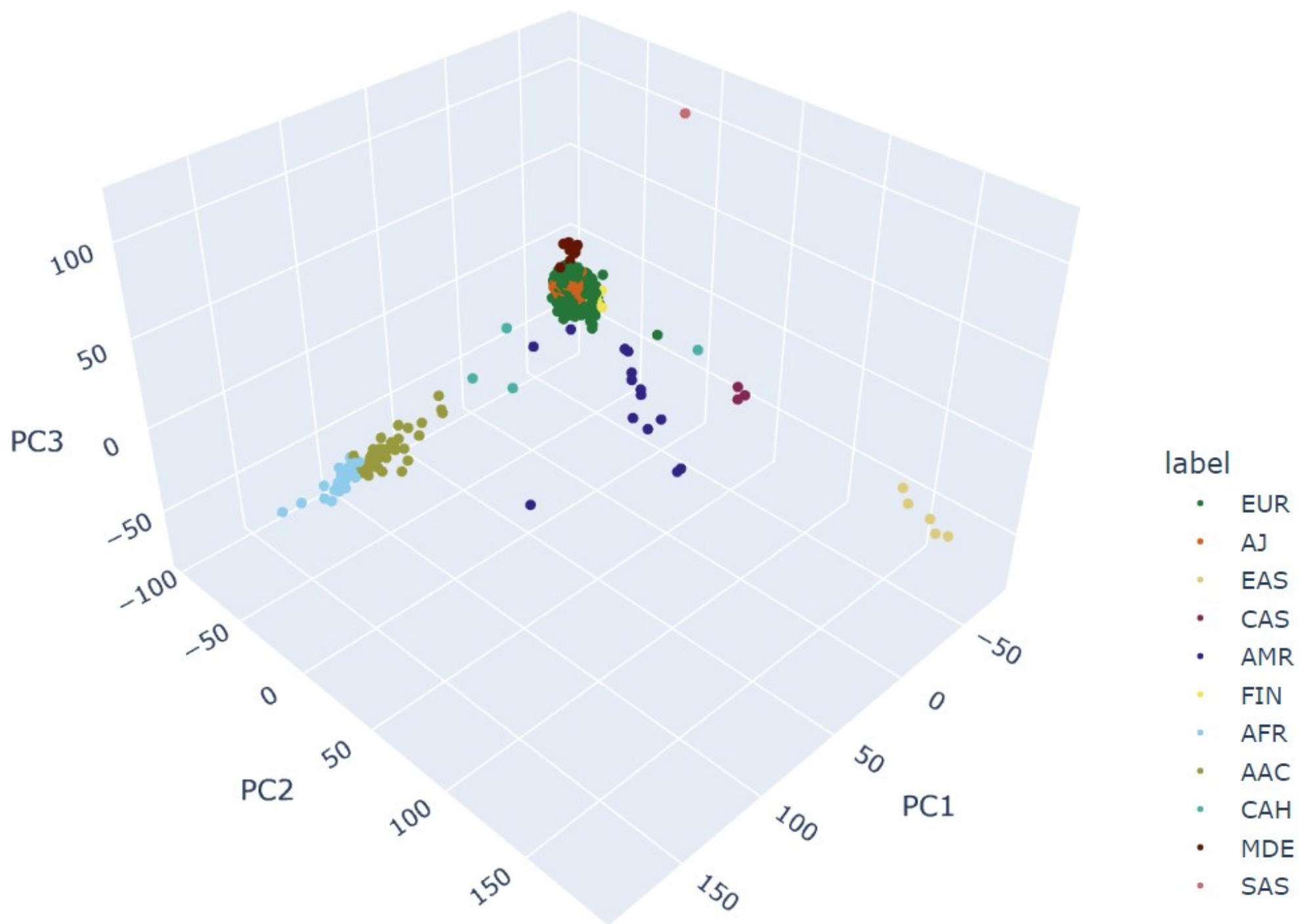

Supplement: Supplement 4 — Supplementary Figure 4- Proportions of individuals carrying both APOE ε4/ε4 genotypes and protective or disease-modifying variants across 11 genetic ancestries in Alzheimer’s disease, related dementias, and controls in all datasets. Supplementary Figures 4A and 4C represent SNP distribution within each cohort, and Supplementary Figures 4B and 4D represent SNP distribution between cohorts. The total populations of each ancestry were used to generate 4A and 4B, while the total numbers of ε4/ε4 carriers for each ancestry were used to generate 4C and 4D. Supplementary Figures 4B and 4D show allele frequency ratios (AD-to-Control, left; Related dementias-to-Control, right) among APOE ε4/ε4 carriers for each of the candidate protective or disease-modifying variant, per ancestry. Warmer colors represent higher frequencies in cases versus controls, while cooler colors represent higher frequencies in controls versus cases, with dark blue (N/A) representing variants not present in either cases or controls. [file media-4.pdf]

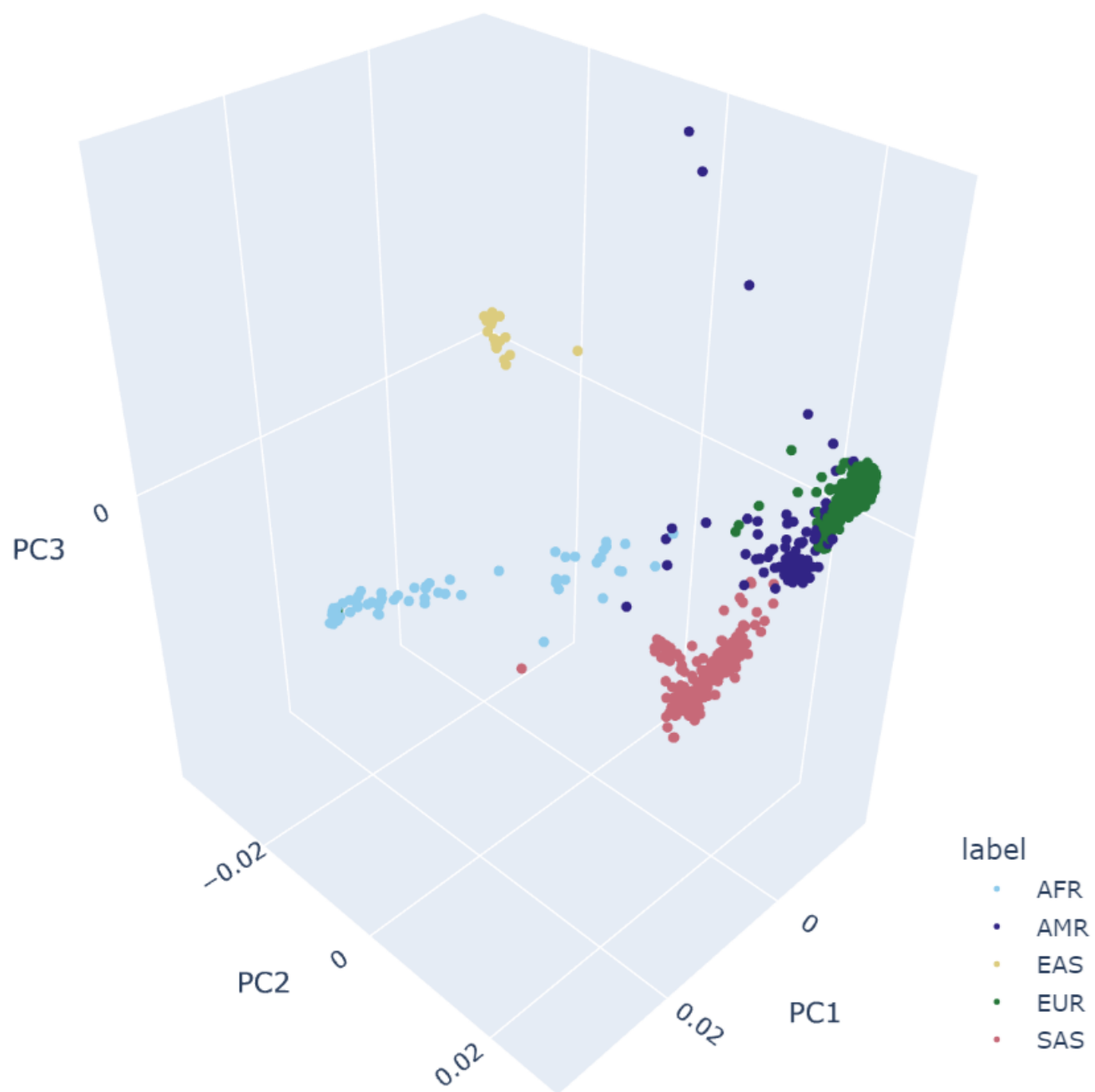

Supplement: Supplement 5 [file media-5.pdf]

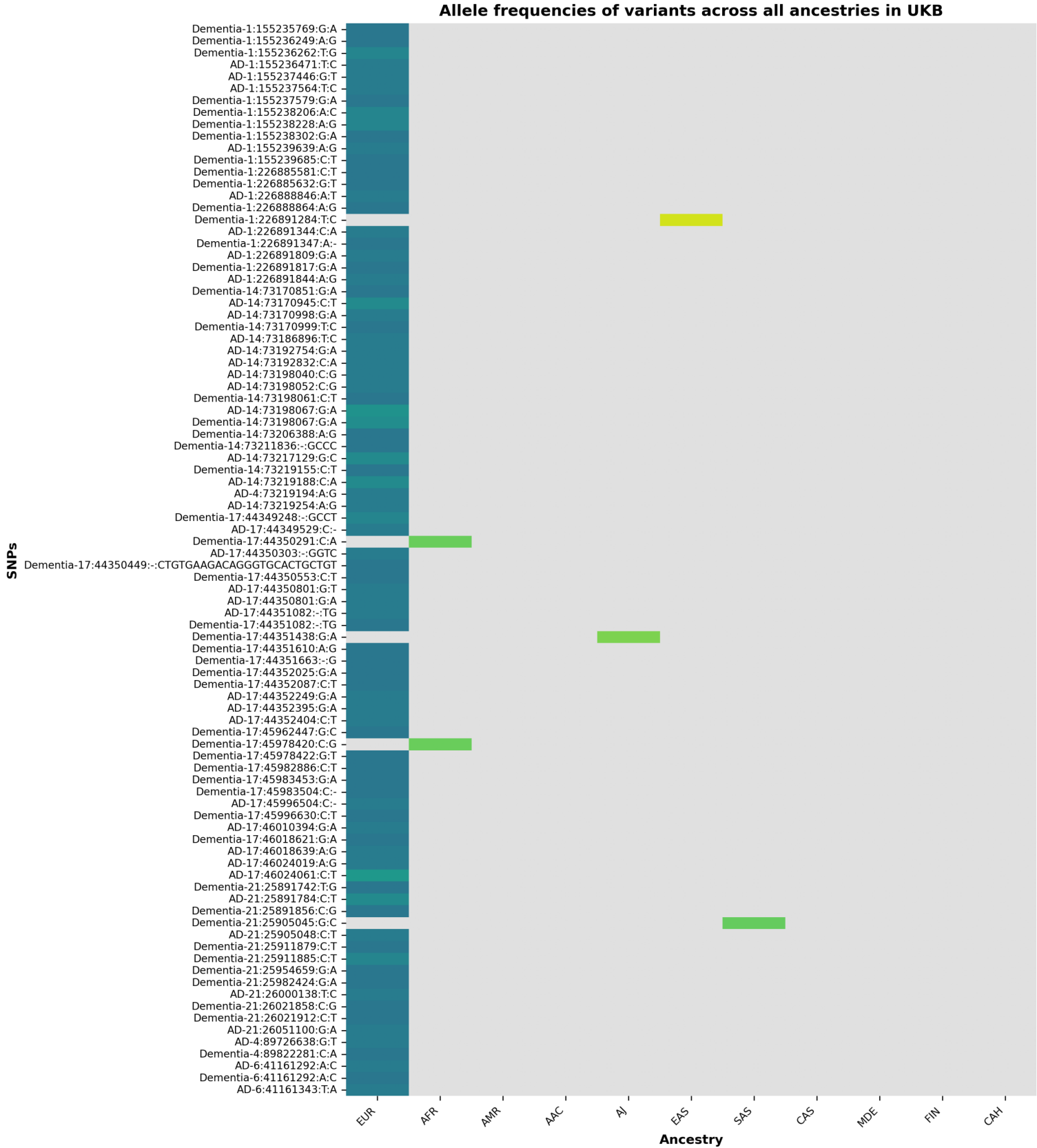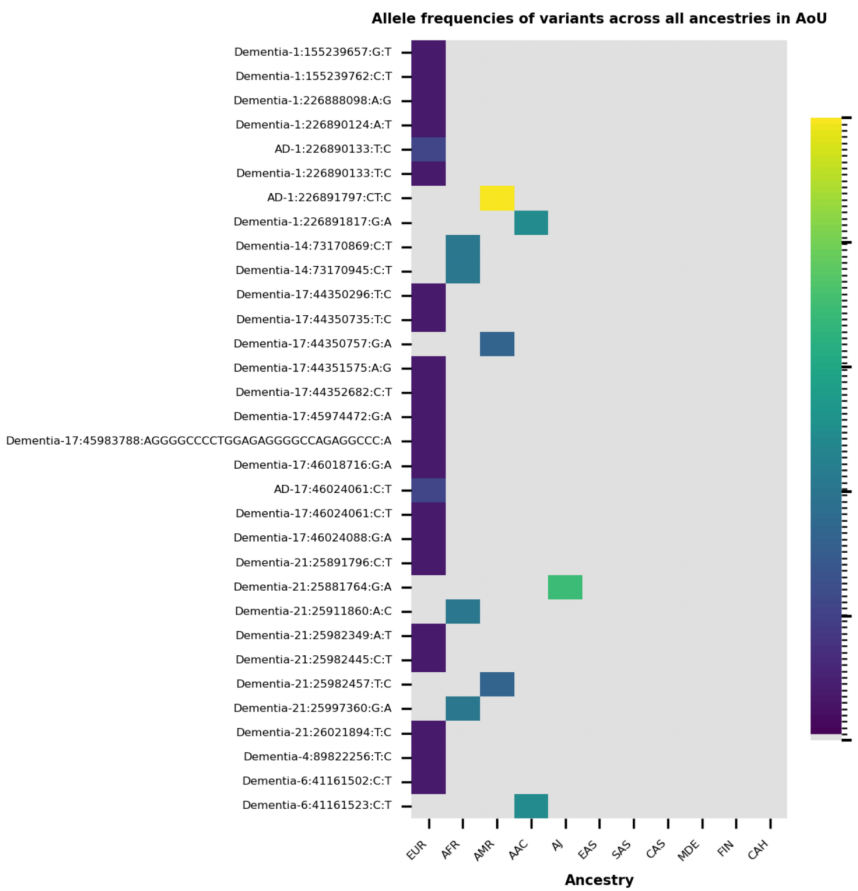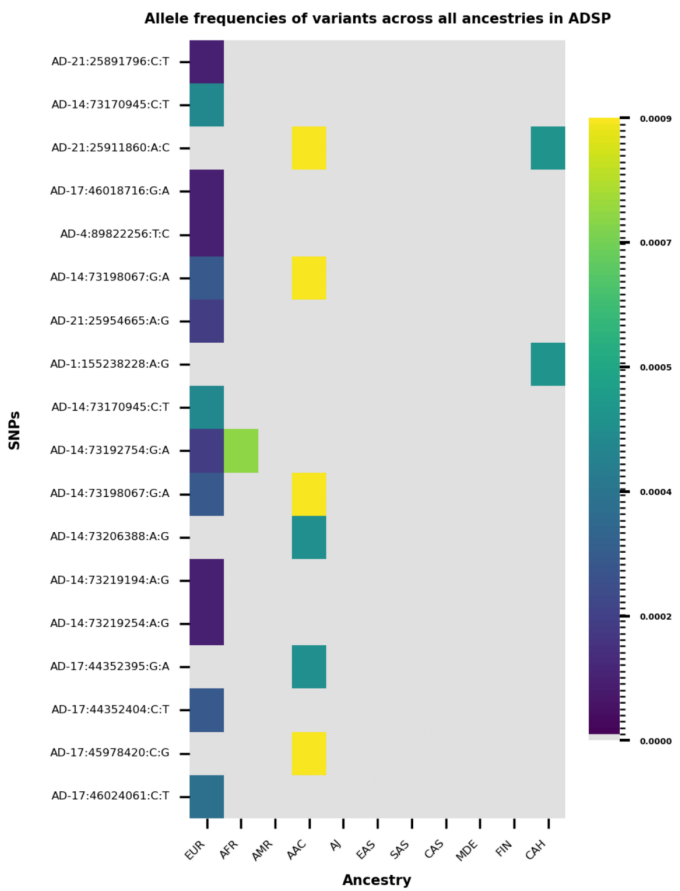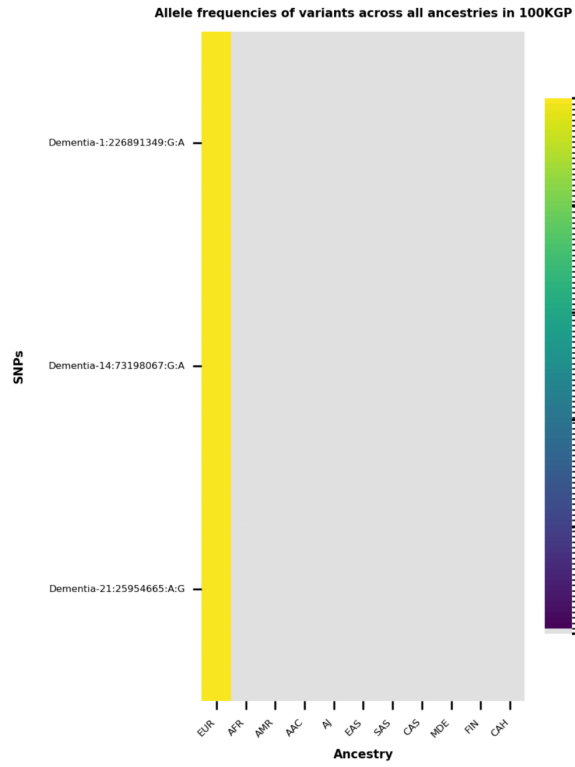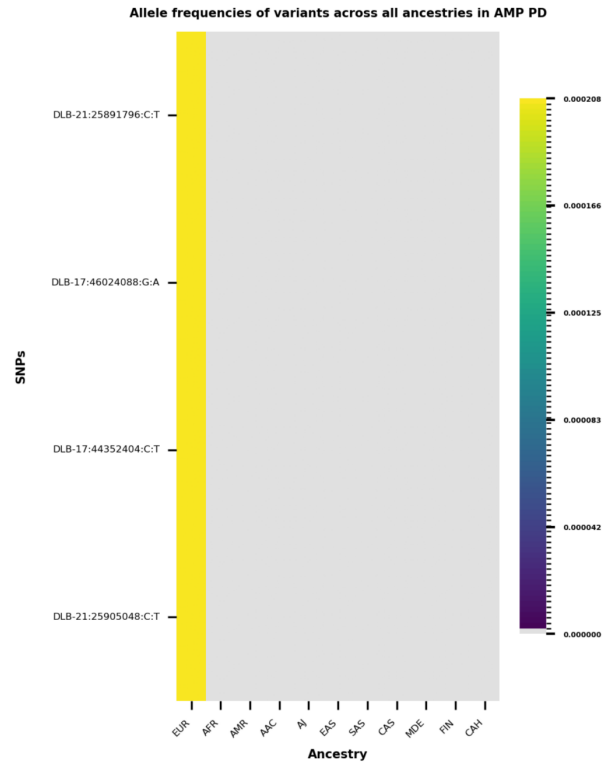

Supplement: Supplement 6 [file media-6.pdf]

Proportions of APOE Genotypes Across Different Ancestries in AD

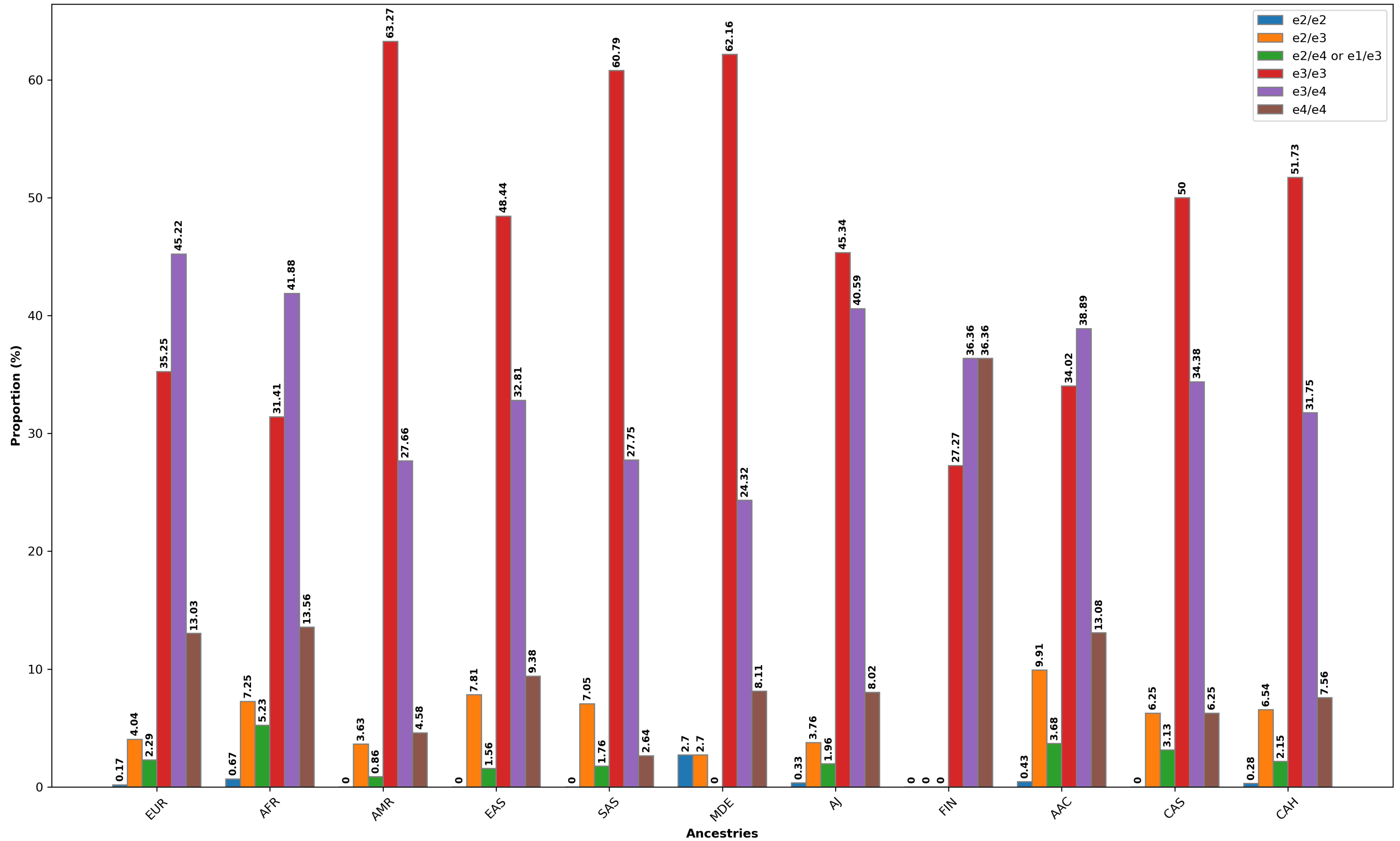

Supplement: Supplement 7 [file media-7.pdf]

Proportions of APOE Genotypes Across Different Ancestries in Related Dementias

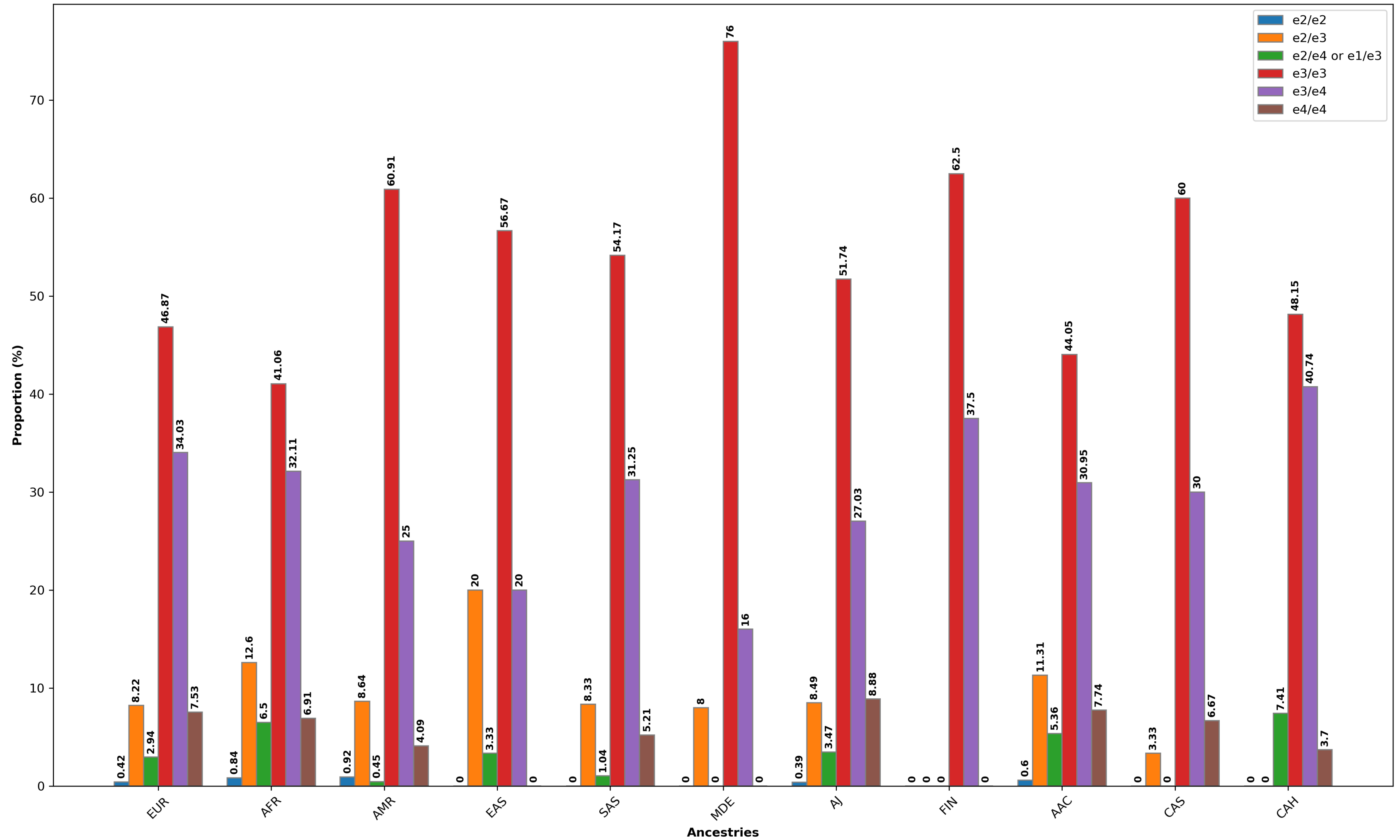

Supplement: Supplement 8 [file media-8.pdf]

Proportions of APOE Genotypes Across Different Ancestries in Controls

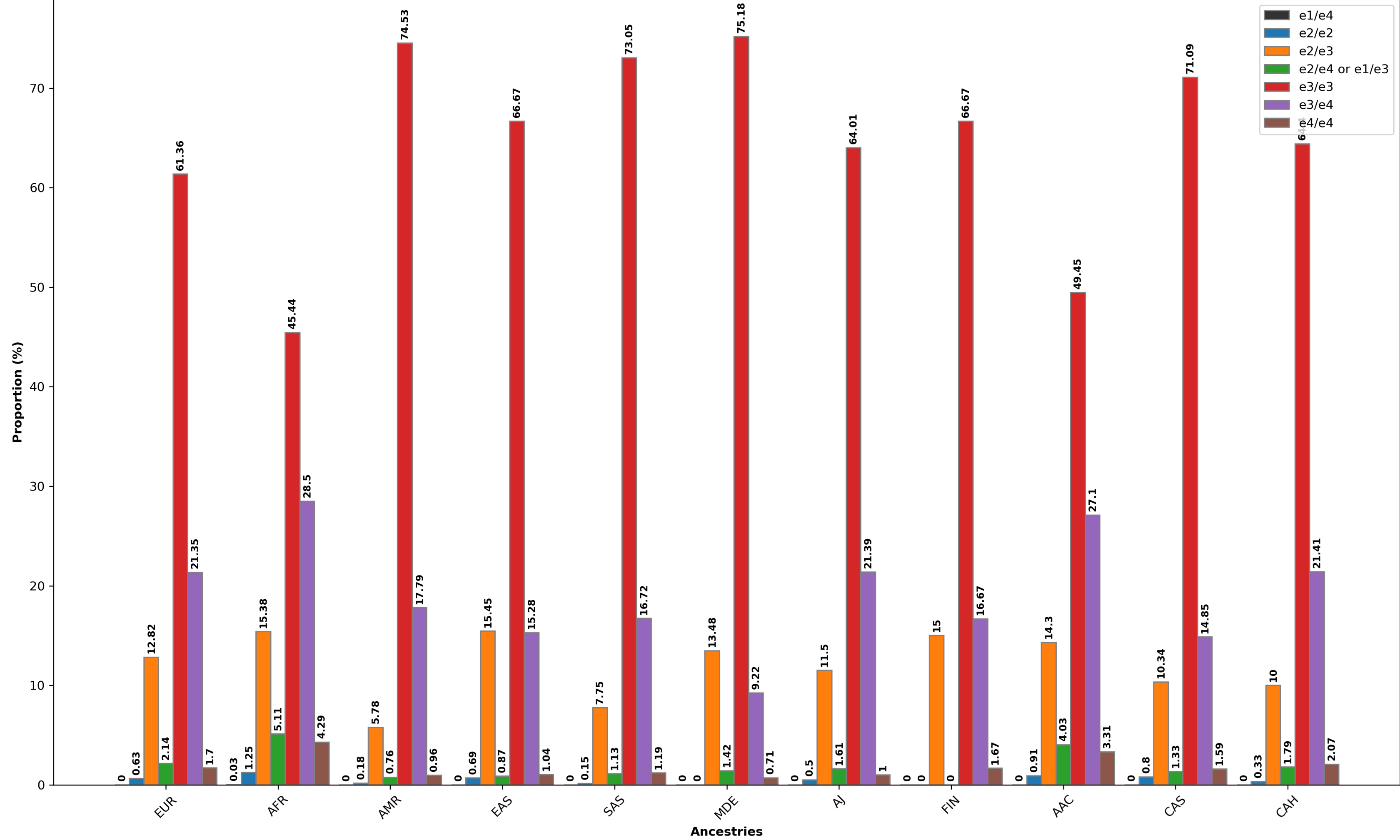

Supplement: Supplement 9 [file media-9.pdf]

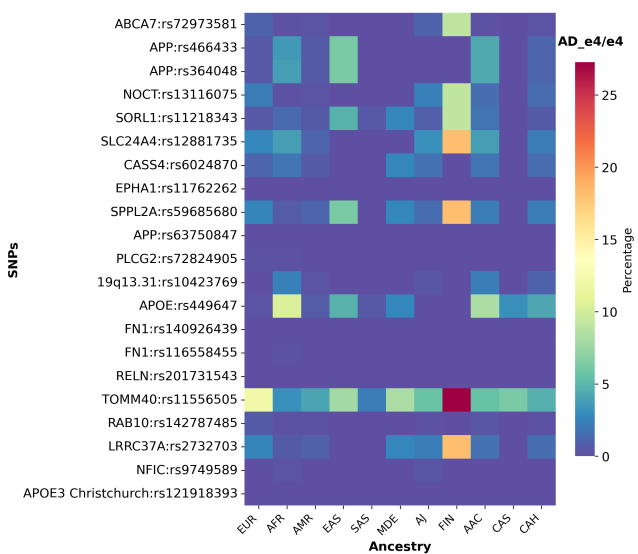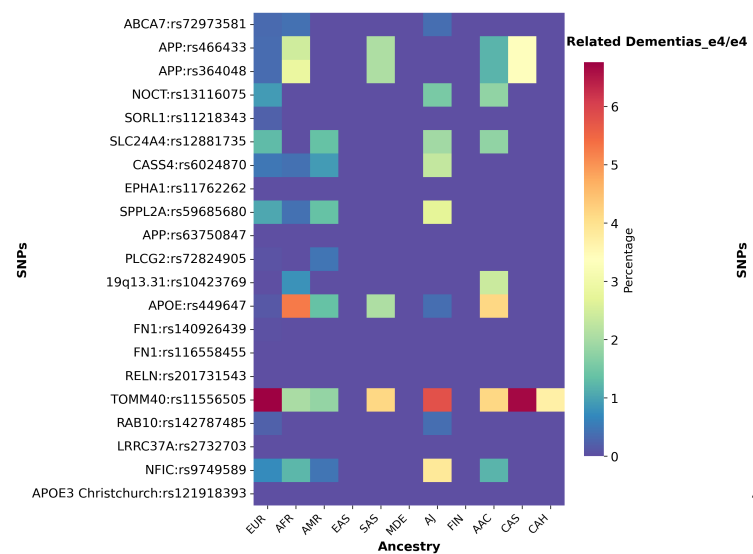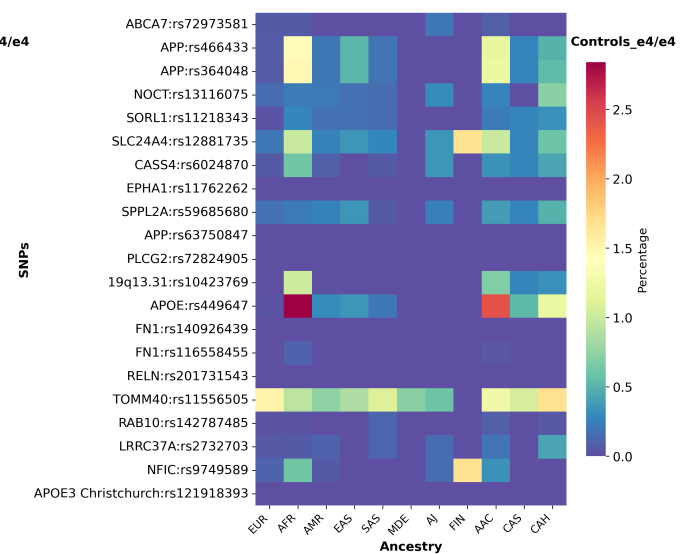

Supplement: Supplement 10 [file media-10.pdf]

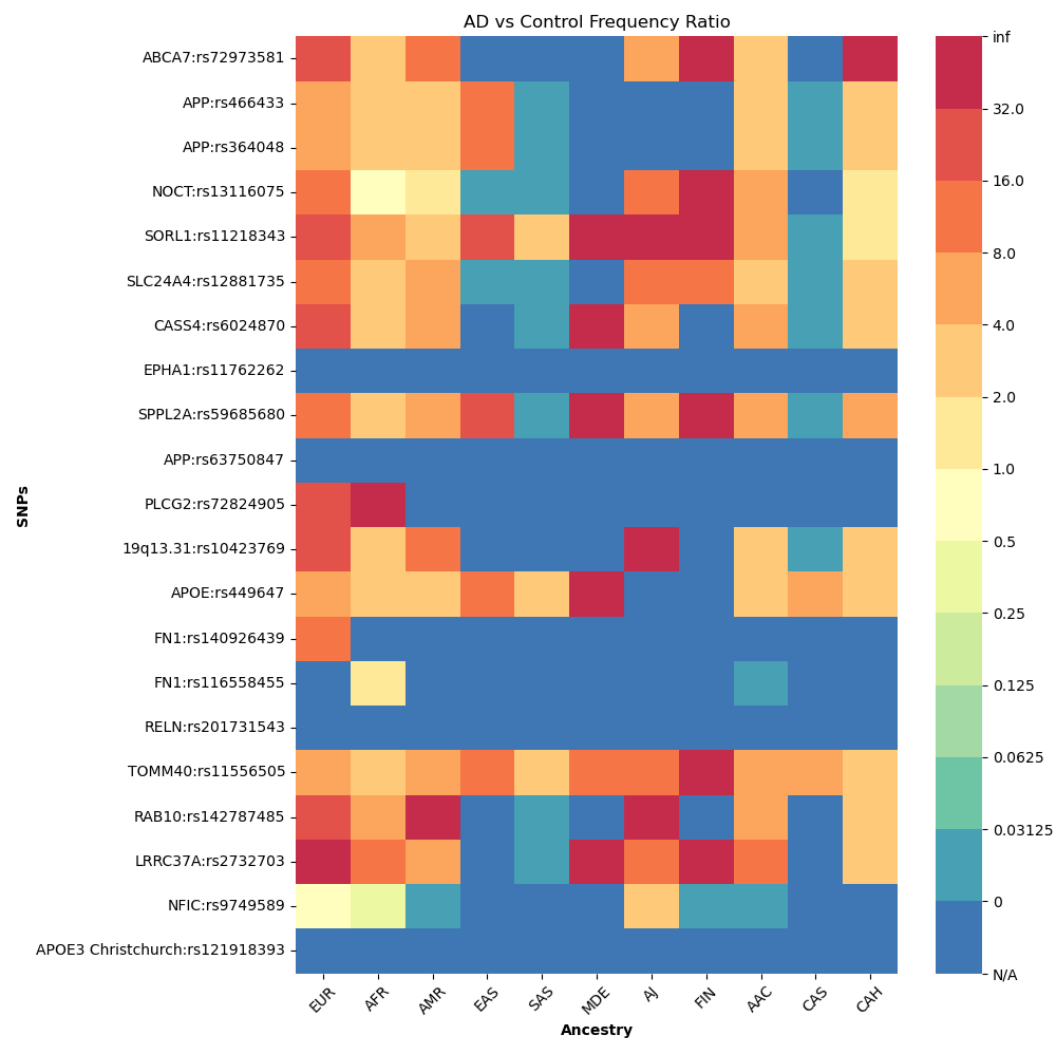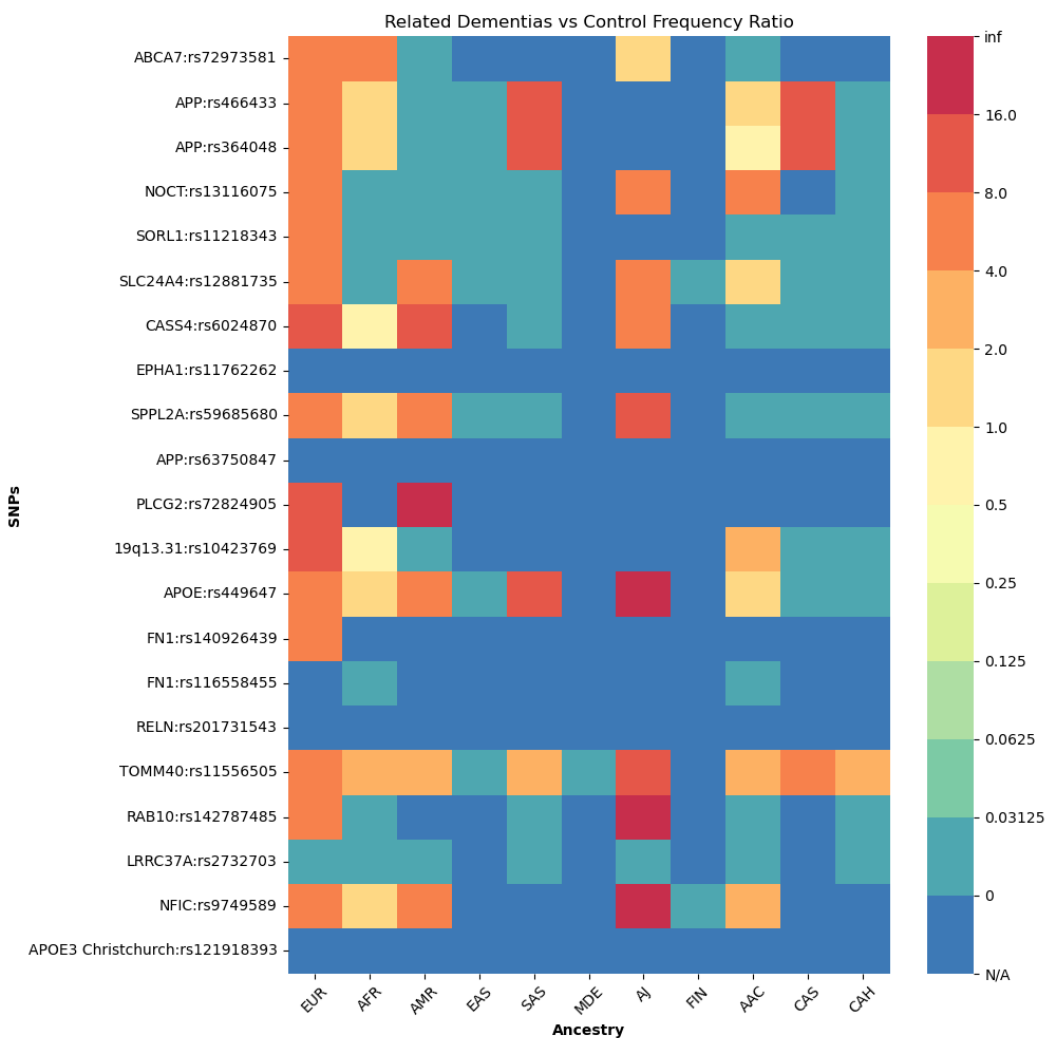

Supplement: Supplement 11 [file media-11.pdf]

SNPs

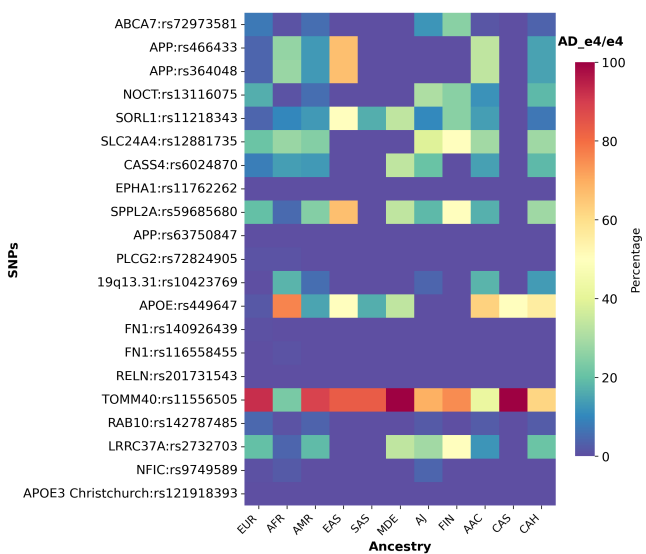

SNPs

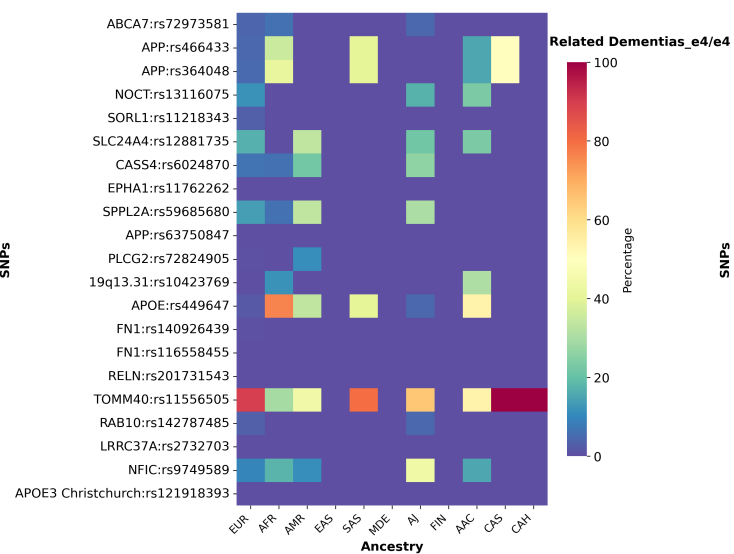

SNPs

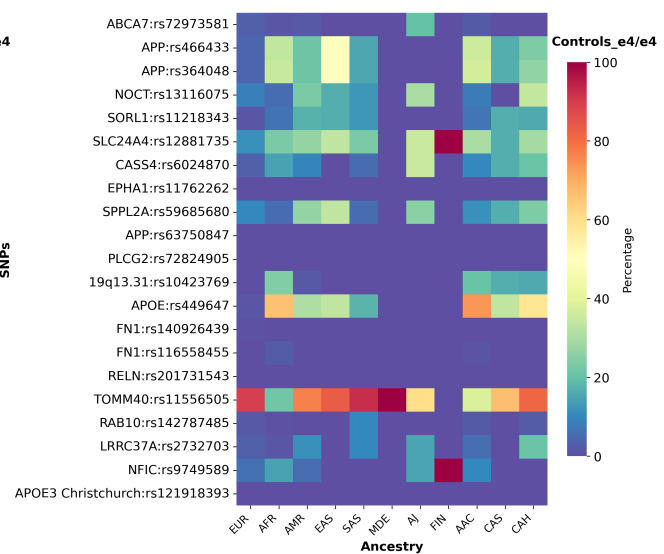

Supplement: Supplement 12 [file media-12.pdf]
