# Supplementary material for: Biobank-scale characterization of Alzheimer’s disease and related dementias identifies potential disease-causing variants, risk factors, and genetic modifiers across diverse ancestries
Source: medRxiv. 2024 Nov 17:2024.11.03.24313587. Preprint. [Version 4] doi: 10.1101/2024.11.03.24313587 (PMC11601747; doi:10.1101/2024.11.03.24313587)
Supplement: Supplement 13 [file media-13.pdf]

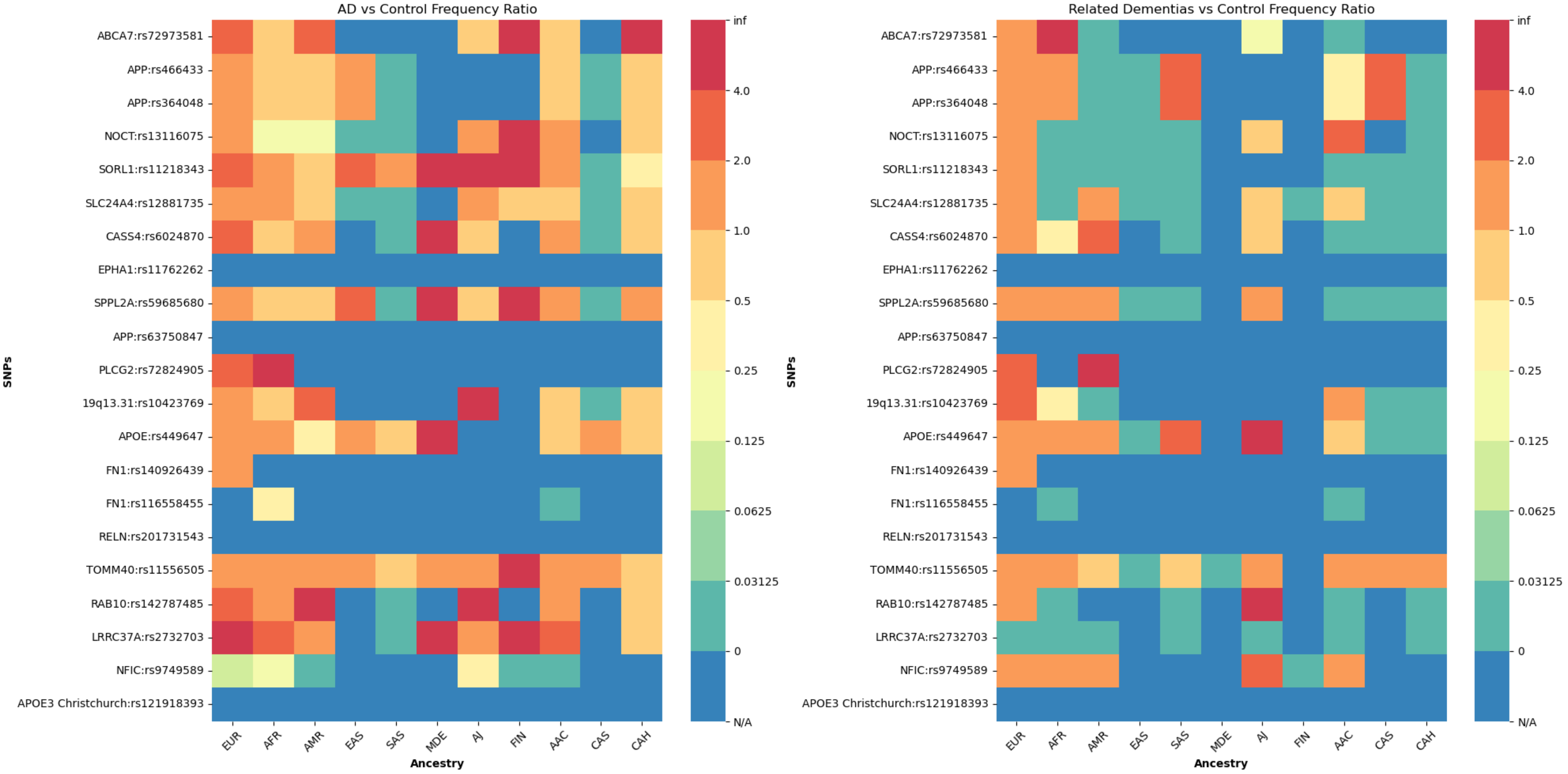

AD vs Control Frequency Ratio
Related Dementias vs Control Frequency Ratio
SNPs
ABCA7:rs72973581
APP:rs466433
APP:rs364048
NOCT:rs13116075
SORL1:rs11218343
SLC24A4:rs12881735
CASS4:rs6024870
EPHA1:rs11762262
SPPL2A:rs59685680
APP:rs63750847
PLCG2:rs72824905
19q13.31:rs10423769
APOE:rs449647
FN1:rs140926439
FN1:rs116558455
RELN:rs201731543
TOMM40:rs11556505
RAB10:rs142787485
LRRC37A:rs2732703
NFIC:rs9749589
APOE3 Christchurch:rs121918393
EUR
AFR
AMR
EAS
SAS
MDE
AJ
FIN
AAC
CAS
CAH
Ancestry
inf
4.0
2.0
1.0
0.5
0.25
0.125
0.0625
0.03125
0
N/A
